# Quantification of myocardial fibrosis by delayed-enhanced MRI in patients with severe aortic valve disease: correlation with quantified histopathology

**DOI:** 10.1186/1532-429X-11-S1-O67

**Published:** 2009-01-28

**Authors:** Carlos E Rochitte, Clério F Azevedo, Marcelo Nigri, Flavio Tarasoutchi, Pablo M Pommerantzeff, Roney O Sampaio, Max Grinberg, Maria L Higuchi

**Affiliations:** Heart Institute – InCor – University of São Paulo Medical School, São Paulo, Brazil

**Keywords:** Aortic Stenosis, Aortic Regurgitation, Myocardial Fibrosis, Aortic Valve Disease, Overt Heart Failure

## Introduction

Chronic aortic valve disease is characterized by progressive accumulation of interstitial myocardial fibrosis (MF) and impairment of myocyte ultrastructure. The amount of interstitial MF may play an important role in the transition from well-compensated hypertrophy to overt heart failure in the setting of chronic LV mechanical overload. However, assessment of interstitial MF and myocyte degeneration has only been possible through histological analyses of myocardial fragments from endomyocardial biopsies. Recently, delayed-enhancement magnetic resonance imaging (deMRI) has been shown to provide an accurate assessment of myocardial necrosis and fibrosis.

## Purpose

We employed a semi-automatic algorithm for the quantification of MF using deMRI technique in patients with severe aortic valve disease. We sought to determine whether the amount of MF by deMRI demonstrated good correlation when compared with the gold-standard histopathological analyses. In addition, we investigated the relationship between the amount of MF and resting LV function before aortic valve replacement (AVR) surgery.

## Methods

Fifty-four patients scheduled to undergo AVR surgery were examined by cine and deMRI in a 1.5 T scanner. From the deMRI dataset, the regions of MF were automatically determined as the sum of pixels with signal intensity (SI) above a pre-determined threshold. The definition of this threshold was based on the mean SI of total myocardium, plus the SI variability we would expect from a non-diseased myocardium (free of focal regions of MF), and also taking into consideration the variability introduced by image noise. More specifically, the threshold was calculated as: mean SI of total myocardium + 2 standard deviations (SD) of mean SI of a remote area + 2 SD of mean SI of air. The remote area was manually delineated in a myocardial region free of hyper-enhancement. In addition, interstitial MF was quantified by histological analysis of myocardial samples obtained during AVR surgery and stained with picrosirius red. Eight subjects with no previous history and who died of noncardiac causes served as controls for the quantitative histopathology.

## Results

The amount of interstitial MF determined by histopathology was higher in patients with aortic valve disease than in controls (24.6 ± 9.8% versus 6.0 ± 1.8%, P < 0.0001). For each patient, the amount of interstitial MF was considered abnormally increased when it was higher than mean+2 SD of control interstitial MF, i.e., higher than 9.6% (6.0% + 3.6%). The amount of MF measured by deMRI was 3.72 ± 2.17% for all patients, 4.35 ± 2.32% in aortic regurgitation and 3.15 ± 1.87% in aortic stenosis subgroup (p = 0.04 between subgroups). Quantification of MF by de-MRI showed good correlation with measurements obtained by histopathology (y = 3.10x + 13.0; r = 0.69, P < 0.0001). Correlation was also good if considered only the subgroup of patients with aortic regurgitation (y = 3.09x + 12.3; r = 0.70, P < 0.0001) or aortic stenosis (y = 3.34x + 12.9; r = 0.67, P = 0.0001). Considering histopathology as the method of reference, ROC analysis revealed a good accuracy of deMRI in identifying patients with increased degrees of MF accumulation (area under the curve = 0.92; 95% CI 0.81 to 1.00). In addition, the amount of MF by deMRI showed an inverse correlation with LV EF (r = -0.67; p < 0.0001) and direct correlations with LV EDV (r = 0.46; p = 0.0005), LV ESV (r = 0.62; p < 0.0001) and LV mass (r = 0.46; p = 0.0005) before AVR surgery.

## Conclusion

Delayed-enhanced MRI allows for the non-invasive quantification of MF with good accuracy when compared with histopathology in patients with severe aortic valve disease. In addition, the amount of MF by deMRI is associated with worse LV morphological and functional parameters before AVR surgery in this population.

